# Dual-Strategy Design of Molecular-Weight-Engineered PEDOT:PSS Complex Films for Enhanced Mechanical Ductility and Environmental Robustness

**DOI:** 10.1021/acsami.5c17154

**Published:** 2025-10-25

**Authors:** Jie-Dong Hu, Jui-Ling Shih, Kuan-Yi Wu

**Affiliations:** Department of Chemical Engineering and Biotechnology, 34877 National Taipei University of Technology, Taipei 10608, Taiwan

**Keywords:** Conductive polymers, PEDOT:PSS, molecular-weight engineering, hydrogen bonding, polymer blend, and wearable electronics

## Abstract

Developing ductile and environmentally robust conductive materials is essential for next-generation wearable electronics, particularly those operating under harsh conditions. Poly­(3,4-ethylenedioxythiophene):poly­(styrenesulfonate) (PEDOT:PSS), a hygroscopic intrinsically conducting polymer, offers high electrical conductivity (σ_e_) and inherent flexibility. However, its multiscale structural defects significantly limit its mechanical deformability across diverse environments. Herein, we propose a dual-strategy design that integrates (1) molecular-weight engineering and (2) hydrogen-bond-driven polymer complexation, achieved by incorporating ultrahigh molecular weight (*M*_w_) poly­(ethylene oxide) (PEO; subzero *T*_g_) into a high-*M*_w_ PEDOT:PSS matrix. It enables the construction of hydrogen-bonded PEDOT:PSS/PEO complex films with enhanced mechanical ductility and environmental tolerance. Structural characterization confirms that H-bonds between PSS and PEO improve miscibility. The involvement of ultrahigh *M*_w_ PEO chains softens the rigid PEDOT:PSS matrix and promotes extensive chain entanglements, yielding films with elongation at break (ε_break_) around 60% while maintaining a high σ_e_ of 100 S·cm^–1^ at 40 wt % PEO. Notably, the flexible PEO chains enable hygroscopic PEDOT:PSS/PEO films to retain the ε_break_ > 30% across a wide temperature range (−20 to 60 °C) or at low-humidity conditions (RH = 10%). In particular, the PEDOT:PSS/PEO films exhibit antifreezing performance, retaining ε_break_ ∼ 42% at −20 °C. These findings demonstrate a synergistic molecular-weight engineering strategy, combined with flexible H-bond complexation to produce ductile and environmentally tolerant conductive films for next-generation wearable electronics.

## Graphical abstract

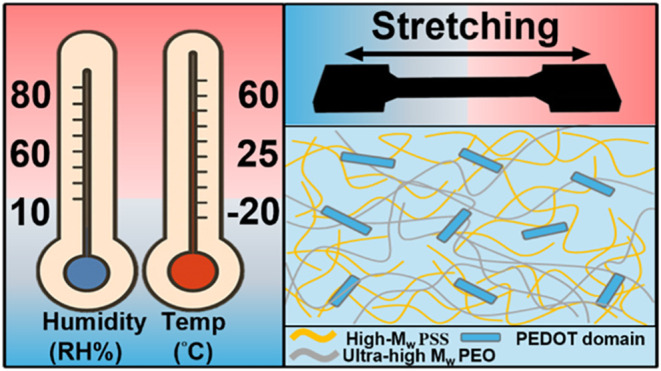

## Introduction

The rapid advancement of flexible electronics in wearable devices has driven increasing demand for conductive materials that can maintain both electrical performance and mechanical ductility.^1,2^ Given that human skin and muscles can undergo strains during movement, it is imperative to develop conductors with sufficient mechanical deformation while maintaining stable electrical performance.^3–5^ In addition, environmental adaptability is another crucial factor, as wearable electronics are often exposed to fluctuations in temperature and humidity.^6,7^ Conductive materials that lack adequate tolerance against environmental stressors are vulnerable to fracture during stretching, which hinders their integration into wearable applications.^8,9^ Consequently, it is essential to develop conductive materials with high mechanical durability across diverse operating conditions.^10,11^

Achieving this balance between electrical conductivity (σ_e_), mechanical properties, and environmental tolerance remains a key challenge in advancing next-generation wearable electronic systems.^10,12^ Among various conductive materials such as metal and carbon materials, the intrinsically conducting polymer, poly­(3,4-ethylenedioxythiophene):poly­(styrenesulfonate) (PEDOT:PSS) stands out for its solution processability and moderate electrical conductivity (σ_e_ > 10^3^ S·cm^–1^).^13,14^ Nonetheless, its rigid polymer backbone, strong H-bond and electrostatic interactions within PEDOT:PSS cause an extremely high glass transition temperature (*T*_g_).^15^ Moreover, PEDOT:PSS naturally assembles into granular micelles at the mesoscales, where PSS-rich shells encapsulate PEDOT-rich cores.^16^ The colloidal aggregation further induces interfacial defects between adjacent micelles.^17–19^ Although the hygroscopic nature of PSS enables water-induced plasticization to the PEDOT:PSS matrix, the inadequate structural hierarchy still makes commercial PH1000 exhibit mechanical brittleness, with elongation at break (ε_break_) of approximately 3%.^20–22^ Consequently, these structural constraints still render it unsuitable for wearable electronics without further modification.

To improve the mechanical properties, several strategies were proposed to modulate the PEDOT:PSS hierarchical morphology.^12,23,24^ One approach employs hydrophilic small-molecule additives, which can plasticize the rigid PEDOT:PSS matrix.^24,25^ Nevertheless, owing to the lack of chain entanglement, these additives yield only a modest improvement in mechanical ductility. In contrast, through molecular-weight (*M*_w_) engineering, the higher *M*_w_ of PSS increases the chain entanglement within the PEDOT:PSS network, thereby giving a larger ε_break_ around 40% for the high-*M*_w_ PEDOT:PSS fibers.^26^ In parallel, blending PEDOT:PSS with hydrophilic polymers is a well-known strategy to reinforce the PEDOT:PSS matrix and enhance ductility.^27^ The intermolecular H-bond interaction facilitates the interpenetration of hydrophilic polymer chains in the PEDOT:PSS morphology. Notably, many hydrophilic polymers with H-bond donor groups also absorb ambient moisture to improve extensibility.^28^ Examples like poly­(vinyl alcohol) (PVA) and poly­(acrylic acid) (PAA) have been employed to enhance the ε_break_ of PEDOT:PSS blend films to nearly 50%, while maintaining a high σ_e_ of approximately 200 S·cm^–1^ under normal ambient conditions.^29–31^

Although the above-mentioned strategies have yielded notable mechanical enhancements under normal ambient conditions,^23,24,26^ their effectiveness often diminishes when PEDOT:PSS-based electronics are exposed to harsher environments. Given the intrinsic hygroscopicity of PEDOT:PSS-based materials, their mechanical behaviors are highly susceptible to humidity and temperature fluctuations. Under dry or high-temperature environments, the inevitable loss of absorbed water stiffens the polymer matrix. It remarkably reduces its mechanical deformability, as dehydration drives the H-bond-donor polymers in PEDOT:PSS-based materials to revert to a glassy state.^32–35^ In addition, to broaden the applicability under subzero temperatures, preventing water crystallization within the hygroscopic PEDOT:PSS morphology becomes crucial for preserving their mechanical ductility. Thus, imparting the antifreezing capability is the key to operating hygroscopic PEDOT:PSS conductors under cold circumstances. To the best of our knowledge, no effective strategy has been reported that simultaneously improves the mechanical ductility of conducting polymers under diverse environments, highlighting a critical knowledge gap.

Given that an ideal structural hierarchy features an entangled polymer network with intrinsically flexible constituents, we synergize the molecular-weight engineering and hydrogen-bond-driven polymer complexation strategies by blending the ultrahigh *M*_w_ poly­(ethylene oxide) (PEO_8000_; *M*_w_ = 8000 kg·mol^–1^) into the high-*M*_w_ PEDOT:PSS (PSS; *M*_w_ = 1000 kg·mol^–1^), as illustrated in Scheme 1.^26^ Unlike many hydrophilic polymers that act as H-bond donors and thus elevate *T*_g_,^32,33^ flexible PEO chains only behave as H-bond acceptors, resulting in a subzero *T*_g_.^36^ Structural characterization reveals that the PEO_8000_ forms H-bond complexes with PSS.^37^ This interaction promotes high miscibility in PEDOT:PSS/PEO_8000_ blends up to [PEO] = 37 wt %, where flexible PEO chains effectively interpenetrate the rigid PEDOT:PSS matrix and enhance mechanical ductility. Besides, at the critical composition of 40 wt %, the excess PEO undergoes crystallization and first gives the heterogeneous morphology with interspherulitic segregation. As [PEO] exceeds 50 wt %, the crystallization-induced phase separation becomes more pronounced, changing to spherulitic impingement throughout the PEDOT:PSS/PEO_8000_ matrix. However, the misorientation in the spherulitic impingement disrupts the PEDOT conductive network. Therefore, an optimal balance between conductivity (σ_e_ = 100 S·cm^–1^) and tensile properties (tensile strength (σ_strength_) ∼ 12 MPa; ε_break_ ∼ 60%) is achieved at [PEO] = 40 wt % under the normal ambient environment (RH = 60%;25 °C). In addition, the mechanical performances of hygroscopic PEDOT:PSS/PEO_8000_ complex films are evaluated under varying environments. In low-humidity (RH = 10%) or high-temperature (*T* = 60 °C) conditions, the dehydrated PEDOT:PSS/PEO_8000_ films show intrinsic mechanical ductility with σ_strength_ > 25 MPa and ε_break_ > 30%. Moreover, intermolecular H-bonds within the hydroscopic PEDOT:PSS/PEO_8000_ blend suppress the crystallization of bound waters, imparting antifreezing properties to the film, enabling excellent ε_break_ ∼ 42% even at subzero conditions. As a result, incorporating ultrahigh *M*_w_ PEO into PEDOT:PSS empowers the development of blend films with superior mechanical durability across wide temperature and RH conditions, making them suitable for wearable electronics designed to withstand diverse environmental challenges.

**Scheme 1:**
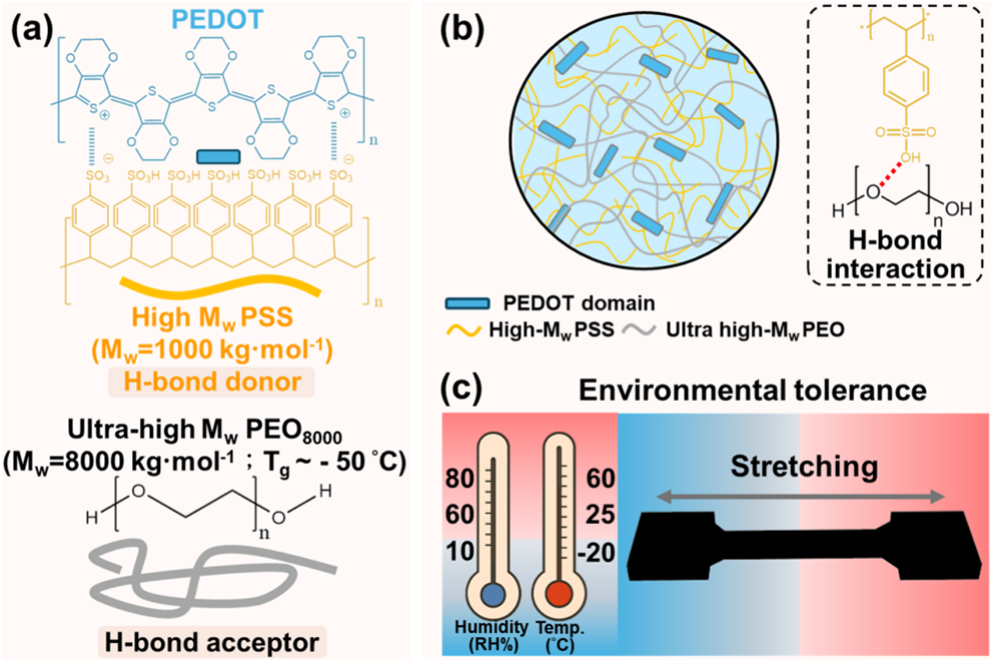
(a) Chemical Structures of the PEDOT:PSS with the High-*M*_w_ PSS (*M*_w_ = 1000 kg·mol^–1^) and Ultrahigh *M*_w_ Poly­(ethylene oxide) (PEO) with *M*_w_ = 8000 kg·mol^–1^; (b) Schematic Illustration of the Entangled PEDOT:PSS/PEO_8000_ Flexible Matrix Driven by H-Bond Complexation of PSS/PEO; and (c) Schematic Illustration of the Mechanical Durability of Ultrahigh *M*_w_ PEDOT:PSS/PEO Conductors under Wide Temperature and Humidity Conditions

## Results and Discussion

### Composition-Dependent Morphology in Ultrahigh *M*_w_ PEDOT:PSS/PEO Blends

The synthetic route of high-*M*_w_ PEDOT:PSS (PSS *M*_w_ = 1000 kg·mol^–1^) is illustrated in Scheme S1, and the ultrahigh *M*_w_ flexible PEO_8000_ (*M*_w_ = 8000 kg·mol^–1^) was selected as the reinforcing component. The fabrication of drop-cast and free-standing PEDOT:PSS/PEO_8000_ films using DMSO as a secondary dopant is described in the Experimental Section.^13^ As a representative amorphous/crystalline polymer blend,^38^ the miscibility between PEDOT:PSS and PEO_8000_ is critical in determining the hierarchical morphology. To investigate the structural evolution across different PEO contents, wide-angle X-ray diffraction (WAXD) was employed to probe the microstructure. In Figure 1a, the WAXD profile of neat PEDOT:PSS exhibits two broad scattering halos at *q*_pss,inter_ = 0.33 Å^–1^ and *q*_pss,intra_ = 1.24Å^–1^, corresponding to an interchain spacing of 18.25 Å and an intrachain distance of 4.94 Å for the PSS segments, respectively.^39^ Meanwhile, the scattering peak at *q*_π–π_ = 1.81 Å^–1^ is referred as the π-π stacking distance of PEDOTs (*d*_π–π_ = 3.46 Å). As the PEO_8000_ content increases to 37 wt %, the WAXD profiles display a gradually intensified amorphous halo in the *q*-range of 0.8–2.2 Å^–1^. This observation indicates that the high miscibility between PEDOT:PSS and PEO_8000_ effectively suppresses PEO crystallization, thereby promoting the formation of the amorphous structure. At [PEO_8000_] = 40 wt %, weak diffraction peaks of PEO crystalline domains emerge, and become more pronounced above 50 wt %. The typical diffractions at *q*_120_ = 1.36 Å^–1^ and *q*_112_ = 1.65 Å^–1^ correspond to the (120) and (112) reflections of crystalline PEO, respectively. These features confirm that excess PEO composition leads to crystallization-induced heterogeneity. Moreover, complementary thermal behavior is evidenced by DSC thermograms (Figure 1b). Neat PEO_8000_ displays a melting endotherm at 68 °C (Δ*H* = 148 J·g^–1^). Upon blending with PEDOT:PSS, the Δ*H* gradually decreases to 3.3 J·g^–1^ at [PEO] = 40 wt % while the *T*_m_ remarkably shifts downward to 56 °C. Below 37 wt %, the *T*_m_ vanishes entirely, reconfirming the formation of a fully amorphous and miscible phase in the PEDOT:PSS/PEO_8000_ blend.

**Figure 1:**
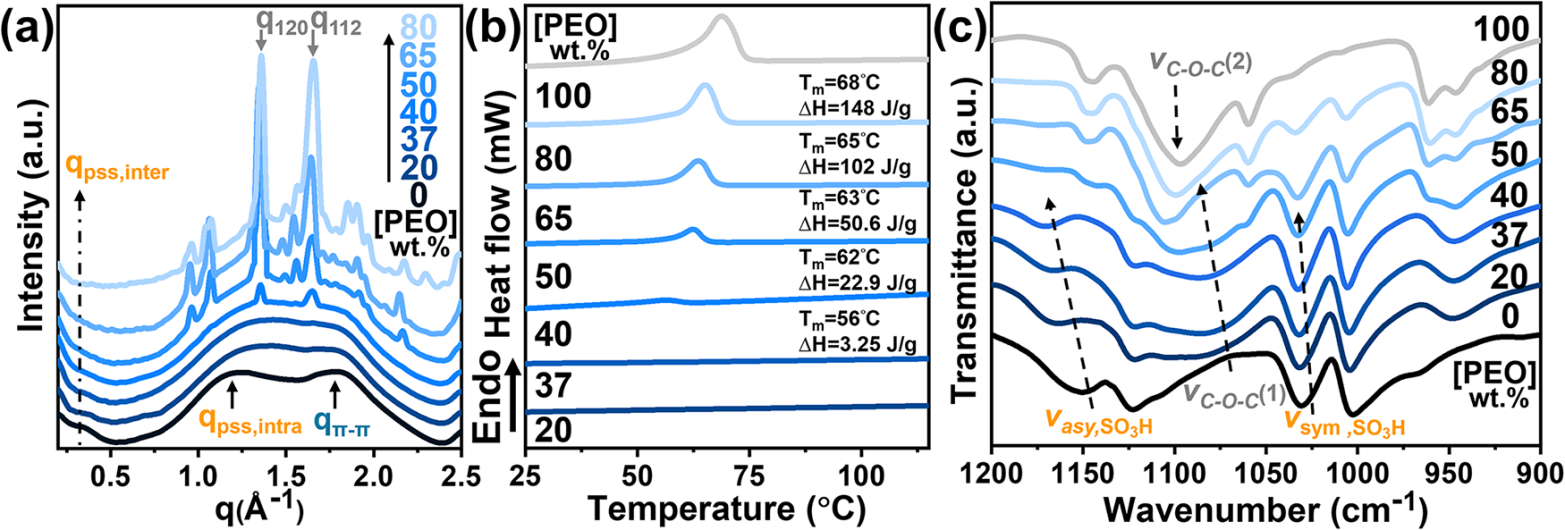
Structural characterization of PEDOT:PSS/PEO_8000_ films: (a) WAXD profiles of PEDOT:PSS/PEO_8000_ blends for various PEO_8000_ content. (b) DSC thermograms of PEDOT:PSS/PEO_8000_ blends. (c) Composition-dependent IR spectrum of PSS/PEO_8000_ blend; ν_*C–O–C*_(1) and ν_*C–O–C*_(2) are the C–O–C stretching vibration of PEOs in the amorphous and crystalline domains, respectively.

The dominant role of intermolecular H-bonds in facilitating the high miscibility of PEDOT:PSS/PEO_8000_ blends is further characterized by FT-IR spectroscopy (Figure 1c). Neat PSS exhibits characteristic stretching bands of −SO_3_H at 1160 cm^–1^ (asymmetric) and 1030 cm^–1^ (symmetric).^15^ Upon increasing PEO content, these bands shift to 1170 and 1033 cm^–1^, respectively; the hypsochromic shifts are associated with the H-bond complexation, where PSS acts as the H-bond donor and PEO as the acceptor. Meanwhile, the C–O–C stretching vibration of amorphous PEO appears at ν_C–O–C_(1) = 1074 cm^–1^ at low PEO content (20 wt %).^40^ As [PEO_8000_] increases to 37 wt %, this band blue-shifts to 1080 cm^–1^, reflecting a reduced average H-bond density surrounding the amorphous PEO chains. At [PEO_8000_] ≥ 50 wt %, the stretching band of C–O–C at ν_C–O–C_(2) = 1100 cm^–1^ from the PEO crystalline domains can be clearly observed,^41^ signifying the crystallization of excess unbound PEO chains due to the insufficient intermolecular H-bonds.^37^ These findings demonstrate that extensive H-bond interactions are the key to the high miscibility of the PEDOT:PSS/PEO blend system.

The above characterization identifies the composition-dependent microstructure of PEDOT:PSS/PEO_8000_ blends, and its corresponding macroscopic morphology was further examined using polarized optical microscopy (POM). Figure S1 shows that neat PEDOT:PSS drop-cast films exhibit an amorphous morphology without birefringence. In contrast, pure PEO_8000_ displays prominent spherulitic impingement with strong birefringence, characteristic of its semicrystalline nature. Upon blending, the film morphology evolves with PEO content. At [PEO_8000_] ≤ 37 wt %, Figure 2 shows that the PEDOT:PSS/PEO blends remain nonbirefringent under POM, reconfirming the homogeneous morphology. At the critical [PEO_8000_] = 40 wt %, a sparse distribution of PEO-rich spherulites with faint birefringence appears, embedded within the amorphous PEDOT:PSS/PEO matrix. Notably, as the PEO content increases beyond 50 wt %, Figure 2 shows enhanced birefringence of PEO-rich spherulites, indicating increased crystallinity. Simultaneously, a higher density of spherulites nucleates and grows, ultimately undergoing impingement with neighboring spherulites.

**Figure 2:**
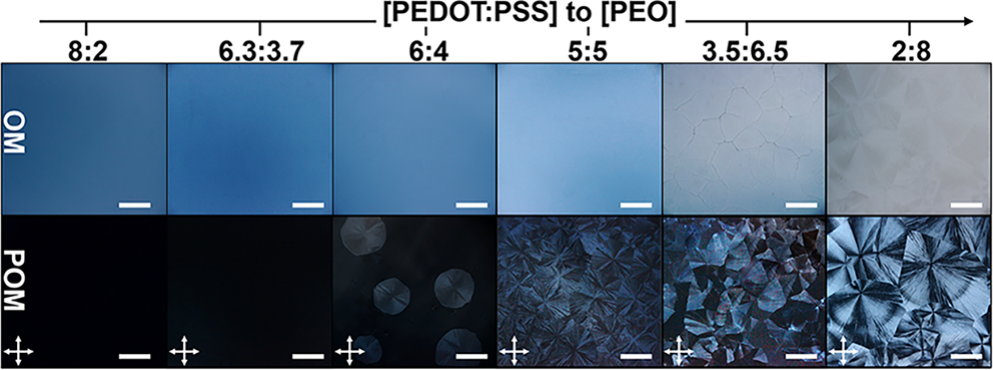
OM and POM micrographs of the PEDOT:PSS/PEO_8000_ drop-cast films with varying PEO mixing ratio. The polarizer and the analyzer (white arrows) are in a perpendicular configuration. Scale bar: 500 μm.

To consolidate this multiscale structural understanding in Figures 1,2, and 3 further presents the schematic of the hierarchical morphology across composition. In the low-PEO regime ([PEO_8000_] ≤ 37 wt %), the blend adopts a homogeneous amorphous morphology, where flexible PEO_8000_ chains interpenetrate the PEDOT:PSS matrix and form the H-bond complex with PSS. Moreover, upon the onset of crystallization at [PEO_8000_] = 40 wt %, a crystallization-driven phase separation leads to the hierarchical heterogeneity. At the macroscopic scale, the interspherulitic segregation gives the formation of PEO-rich spherulites dispersed within a continuous amorphous PEDOT:PSS/PEO matrix; within each PEO-rich spherulitic domain, the interlamellar segregation leads to the radially aligned PEO lamellae crystals interlacing with amorphous PEDOT:PSS/PEO regions.^42–44^ At higher PEO loadings ([PEO_8000_] ≥ 50 wt %), sufficient PEO contents make the spherulitic impingement dominate the morphology with clear boundaries. In spherulitic impingement, the ultralong PEO_8000_ chains act as tie molecules to bridge adjacent spherulites, resulting in a continuous semicrystalline network.^45^ However, the mismatch in crystalline orientation at the spherulite interfaces would disrupt the continuous PEDOT-based percolation network.

**Figure 3:**
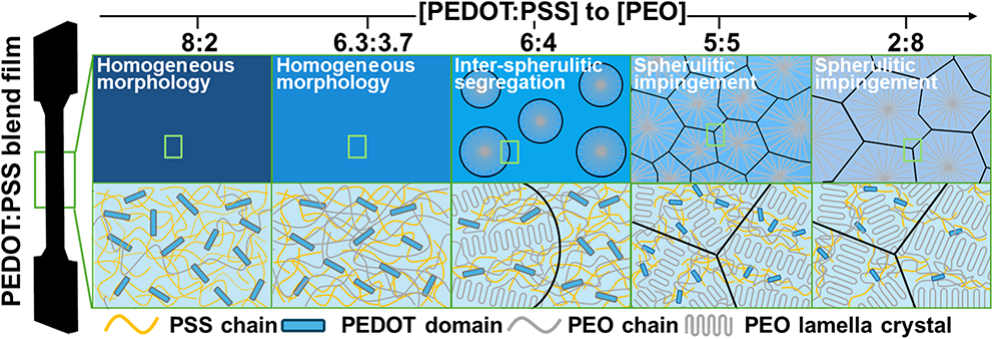
Schematic illustration for the composition-dependent hierarchical morphology of the ultrahigh *M*_w_ PEDOT:PSS/PEO_8000_ blends.

In addition to H-bond interactions, the influence of *M*_w_ on the miscibility of crystalline/amorphous polymer blends is also evaluated. In general, longer polymer chains reduce the entropy of mixing, thereby thermodynamically disfavoring phase miscibility.^46^ Thus, we examined the miscibility behavior of PEDOT:PSS with a series of PEOs of different *M*_w_: PEO_100_, PEO_600_, PEO_1000_, and PEO_3500_. The WAXD profiles in Figure S2 display that all PEDOT:PSS/PEO blends maintained amorphous morphologies at [PEO] ≤ 37 wt %, regardless of PEO *M*_w_. As the PEO content exceeds 40 wt %, the diffraction peaks of crystalline PEO emerge in all samples. These results suggest that intermolecular H-bond is the dominant factor governing miscibility in PEDOT:PSS/PEO blends. Therefore, the ultrahigh *M*_w_ PEOs not only exhibit excellent compatibility with PEDOT:PSS, but their high chain lengths also promote chain entanglement within the polymer matrix, illustrated in Figure 3.

### Dependence of Conductivity/Tensile Behavior on PEDOT:PSS/PEO Composition

The composition-dependent morphology of ultrahigh *M*_w_ PEDOT:PSS/PEO_8000_ complexes (Figure 3) further determines the mechanical and conductive properties. Given that most reported PEDOT:PSS performances were estimated under normal ambient conditions,^24^ these performances were initially evaluated under *T* = 25 °C and RH = 60%. As shown in Figure 4(a–c), neat PEDOT:PSS films exhibit mechanical brittleness with a limited ε_break_ ∼ 5.4%. In contrast, Figure S3 reveals that the PEO_8000_ film exhibits highly ductile behavior, reaching up to ε_break_ above 500%. This pronounced ductility is attributed to the highly entangled semicrystalline morphology of PEO_8000_, where the soft amorphous phase enables large-scale chain mobility during deformation.^47^ Upon increasing [PEO], the well-distributed PEO chains soften the rigid PEDOT:PSS matrix, as supported by the suppressed *T*_g_ (Figure S4). Figure 4(a–c) reveals that enhanced chain mobility improves film ductility, yielding ε_break_ of 48.3% accompanied by reduction in Young’s modulus at [PEO] = 37 wt %. Furthermore, when [PEO] ≥ 40 wt %, PEO crystallization within the PEDOT:PSS/PEO_8000_ films creates crystalline domains that act as physical cross-links, further enhancing mechanical ductility, where ε_break_ reaches around 60% at 40 wt % and exceeds 100% for [PEO] ≥ 50 wt %. Moreover, at [PEO] ≥ 50 wt %, the tensile behavior resembles neat PEO_8000_ films, exhibiting a distinct yield point. This characteristic is primarily attributed to the impinged spherulitic morphology (Figure 3), similar to the observation in neat PEO_8000_ film (Figure S1).

**Figure 4:**
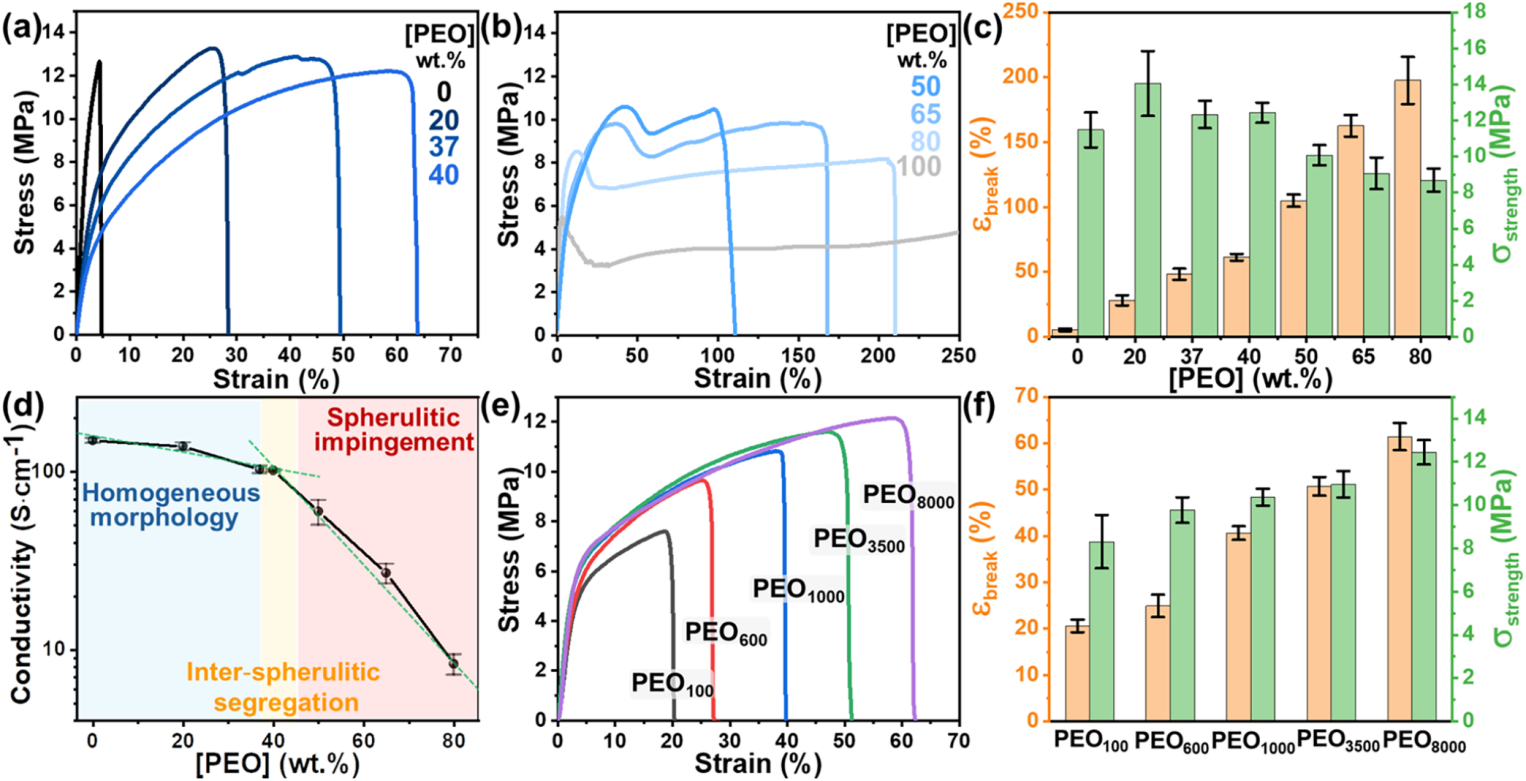
(a, b) Strain–stress curves of the PEDOT:PSS/PEO_8000_ films with varying [PEO] at normal ambient conditions (RH = 60%; *T* = 25 °C). (c) Corresponding tensile strength and elongations. (d) σ_e_ of the PEDOT:PSS/PEO_8000_ films as a function of [PEO]. (e) Strain–stress curves of the PEDOT:PSS/PEO films with varying PEO *M*_w_ (100 kg·mol^–1^ → 8000 kg·mol^–1^) at [PEO] = 40 wt %. (f) Corresponding tensile strengths and elongations. Note: Data are expressed as mean ± SD from five independent samples (*n* = 5).

Although the ultrahigh *M*_w_ PEO significantly enhances the film ductility, increasing the insulating PEO contents also dilutes the conductive network. As shown in Figure 4d, the conductivity of PEDOT:PSS/PEO_8000_ films gradually decreases from 150 to 100 S·cm^–1^ as the insulating [PEO] increases to 40 wt %. However, when [PEO] exceeds 50 wt %, σ_e_ drops sharply below 60 S·cm^–1^. This abrupt decline results from the impinged spherulitic morphology, where the misorientation of adjacent spherulites further hinders the PEDOT conductive network at the boundary, as illustrated in Figure 3. Therefore, at [PEO] = 40 wt %, the PEDOT:PSS/PEO_8000_ blend achieves an optimal trade-off between electrical conductivity (σ_e_ ∼ 100 S·cm^–1^) and mechanical performances (σ_strength_ ∼ 12 MPa; ε_break_ ∼ 60%).

In addition, the *M*_w_ of PEO significantly influences the mechanical performance of PEDOT:PSS/PEO blends. Figure 4(e,f) reveal that at [PEO] = 40 wt %, increasing the *M*_w_ of PEO (100 kg·mol^–1^ → 8000 kg·mol^–1^) leads to an enhancement in ε_break_ from around 20% to 60% and an increase in σ_strength_ from 8.3 to 12.4 MPa. This mechanical improvement confirms that the molecular-weight engineering strategy enables the high-*M*_w_ PEO chains to promote greater chain entanglement and physical reinforcement within the PEDOT:PSS/PEO matrix.

### Temperature–Humidity Dependence of the Ultrahigh *M*_w_ PEDOT:PSS/PEO Electrical and Tensile Behaviors

One of the primary challenges in integrating PEDOT:PSS-based materials into wearable electronics is ensuring reliable electrical output under deformation, ranging from normal to extreme conditions. The hygroscopic nature of PEDOT:PSS allows moisture uptake to soften the rigid polymer matrix, improving ductility under ambient conditions but making it highly sensitive to humidity and temperature changes. As a higher ε_break_ can preserve the integrity of the conductive network during stretching, maintaining ε_break_ across diverse environmental conditions is essential for delivering stable electrical output. Thus, the electrical and tensile behaviors were assessed under varying RH levels and temperatures, as illustrated in Figure 5a.

**Figure 5:**
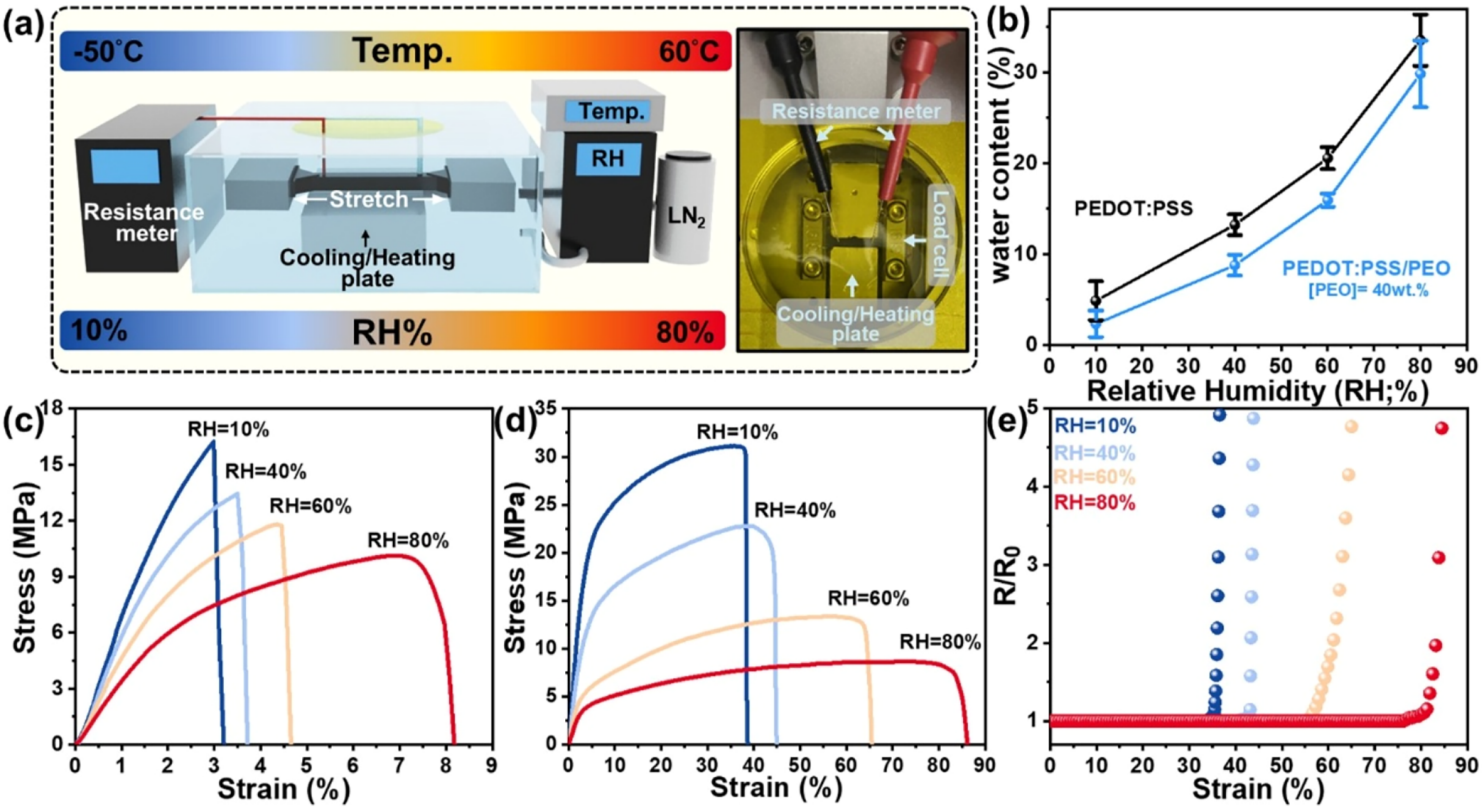
(a) Schematic illustration of the *in situ* tensile and electrical resistance measurements of PEDOT:PSS-based films under controlled humidity and temperature environments. (b) Water content in the PEDOT:PSS-based films at different RH levels under 25 °C. (c, d) Strain–stress curves of (c) PEDOT:PSS and (d) PEDOT:PSS/PEO_8000_ films at different RH levels under 25 °C. (e) Resistance variations of PEDOT:PSS/PEO_8000_ films. R/R_0_ is the value of final resistance/initial resistance. Note: the PEO content is 40 wt %.

Figure 5b first illustrates the moisture uptake of PEDOT:PSS-based films in the RH levels from 10% to 80% under 25 °C. Compared to the neat PEDOT:PSS film, the PEDOT:PSS/PEO_8000_ blends exhibit lower hygroscopicity, attributed to the PEO crystallization. The stress–strain curves in Figure 5(c,d) and the statistical bar charts (Figure S5) further demonstrate the humidity-dependent tensile behaviors at 25 °C. At RH = 80%, both films exhibit the enhanced ε_break_, reaching around 8% for PEDOT:PSS and 84% for PEDOT:PSS/PEO_8000_ films. This improvement results from the water-induced plasticization. As RH decreases from 80% to 10%, the gradual evaporation of water reduces chain mobility, leading to a decrease in ε_break_ while increasing σ_strength_. Under near-dry conditions (RH = 10%), neat PEDOT:PSS films display mechanical brittleness (ε_break_ = 3.2%), whereas PEDOT:PSS/PEO_8000_ films still retain considerable ε_break_ of 38.2%. Without water plasticization, film ductility is predominantly governed by the intrinsic chain mobility. Thus, incorporating ultrahigh *M*_w_ PEO with *T*_g_ around −50 °C (Figure S4) inherently enhances the PEDOT:PSS mechanical deformability, even in dry environments. Moreover, it is important to estimate the electrical resistance variation (*R*/*R*_0_) of PEDOT:PSS/PEO_8000_ films under stretching at different RH levels, shown in Figure 5e. Initially, *R*/*R*_0_ remains nearly unchanged, indicating stable electrical output. However, the electrical resistance exhibits a pronounced rise as the strain is near the ε_break_ shown in Figure 5(d, e). The point where the derivative dR/dε sharply increases can thus be defined as the critical strain (ε*). This abrupt increase in resistance would result from the formation of microcracks and ruptures near the ε_break_, as evidenced by the OM images (Figure S6).^48,49^ Notably, even at low RH = 10%, the dehydrated PEDOT:PSS/PEO_8000_ films can retain ε* > 30%, underscoring the importance of maintaining mechanical ductility to preserve the integrity of the conductive network and ensure stable electrical performance.

In addition, the temperature-dependent mechanical behavior of PEDOT:PSS-based films was systematically investigated, as both water content and phase behavior are sensitive to temperature. As shown in Figure 6a, the PEDOT:PSS/PEO_8000_ films retain notable ductility across subzero to high-temperature ranges, whereas neat PEDOT:PSS films merely exhibit brittleness (Figure S7). To elucidate the high-temperature deformation behavior, temperature-resolved WAXD patterns of PEDOT:PSS/PEO_8000_ films (Figure 6b) reveal a gradual decrease in scattering intensity at *q* ∼ 1.9 Å^–1^ during heating to 60 °C. This attenuation is attributed to progressive evaporation of bound water (Table S1), driven by the thermal weakening of H-bond interactions between water and the polymer matrix.^39^ Despite this dehydration, the stress–strain curves (Figure 6c) and the statistical bar charts (Figure S8) of the PEDOT:PSS/PEO_8000_ films reveal only a modest reduction in ε_break_ to 32.9% at 60 °C. These results reconfirm that the PEDOT:PSS/PEO_8000_ matrix possesses intrinsic stretchability even in thermally perturbed and low-moisture conditions. In contrast, Figure S7 reveals that rigid PEDOT:PSS films at 60 °C are extremely brittle with ε_break_ values dropping to 1.9%. In addition, we also utilize the common hydrophilic polymers, PVA and PAA, as a comparative strategy for mechanical enhancement.^23,29^ Figure S9 reveals that both PEDOT:PSS/PVA and PEDOT:PSS/PAA blends can exhibit a moderate ε_break_ around 33% at ambient conditions. However, under RH = 10% or *T* = 60 °C, water desorption significantly compromises the ductility of these blend films, with ε_break_ decreasing to around 5%. This decline is primarily ascribed to the intrinsic rigidity of the dehydrated PEDOT:PSS/PVA and PEDOT:PSS/PAA matrix since PVA and PAA with −OH or −COOH functional groups also exhibit a relatively high *T*_g_ ∼ 75 °C and 130 °C, as confirmed in Figure S10.

**Figure 6:**
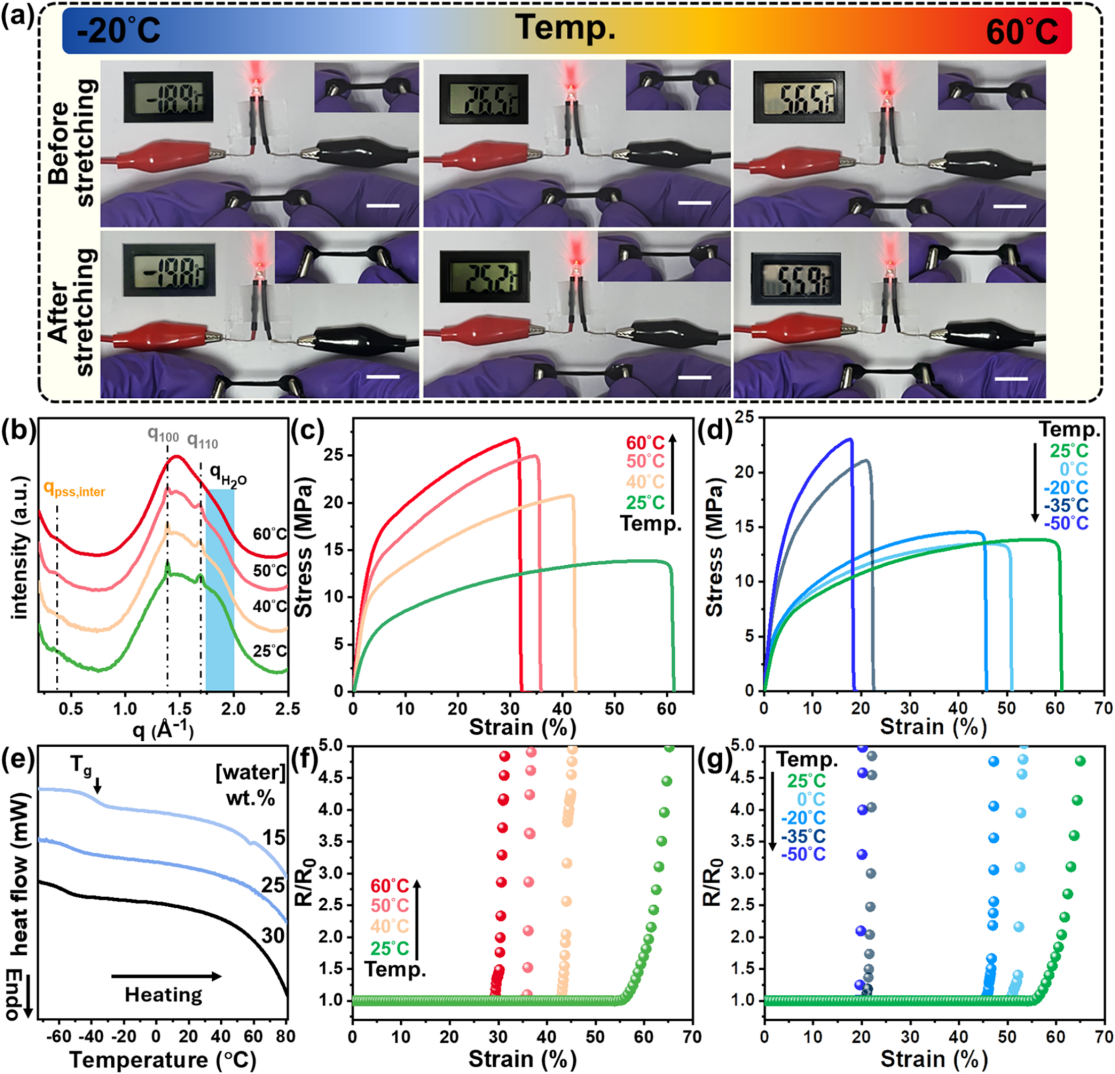
(a) Photographs of the PEDOT:PSS/PEO_8000_ films under stretching at *T* ∼ 60, 25, and −20 °C. Scale bar is 1 cm. (b) Temperature-dependent WAXD profiles of PEDOT:PSS/PEO_8000_ films. (c, d) Strain–stress curves of PEDOT:PSS/PEO_8000_ films from *T* = −50 to 60 °C. (e) Corresponding DSC thermograms of the hydrated PEDOT:PSS/PEO_8000_ film with varying water contents. (f, g) Resistance variations of PEDOT:PSS/PEO_8000_ films from −20 to 60 °C. Note: PEO content is 40 wt %.

In subzero temperature conditions, suppressing ice crystallization is essential to maintaining the mechanical ductility of the hygroscopic PEDOT:PSS/PEO_8000_ films. The PEDOT:PSS/PEO_8000_ films were first equilibrated to a water content of 15 wt % at normal ambient conditions (Figure 5b). Subsequently, the hydrated films were rapidly cooled to assess low-temperature tensile properties. Figure 6(d,e), together with the statistical bar charts in Figure S8, reveal that the hydrated films show antifreezing capability and retain considerable ε_break_ of 42.1% even at −20 °C. Upon cooling to −35 °C, however, the ε_break_ sharply decreases to below 22%; meanwhile, Young’s modulus rises from 347 to 418 MPa (Figure 6d), consistent with the observation of *T*_g_ around −35 °C in Figure 6e. This stiffening transition reflects reduced segmental mobility in the PEDOT:PSS/PEO_8000_ matrix. To further characterize the antifreezing window, DSC analysis (Figure 6e) was also performed at water contents up to 30 wt % according to Figure 5b; no detectable ice melting in the hydrated PEDOT:PSS/PEO_8000_ films was observed. This suggests that the H-bond interactions between bound waters effectively suppress the frozen water. Moreover, temperature-dependent stretchability directly influences the resistance-strain response, illustrated in Figure 6(f,g). Notably, the ε* remains above 30% across −20 to 60 °C, echoing the structural extensibility of ultrahigh *M*_w_ PEDOT:PSS/PEO films (Figure S11). As a result, these findings demonstrate that combining molecular-weight engineering with flexible hydrophilic polymer blending enhances the ductility and environmental robustness of PEDOT:PSS-based materials, offering a viable pathway toward next-generation soft electronics.

## Conclusion

This study combines the molecular-weight engineering and blending strategy to fabricate ultrahigh *M*_w_ PEDOT:PSS/PEO complex films with enhanced mechanical ductility and environmental tolerance. Structural analyses reveal that the intermolecular H-bonds between PSS and PEO ensure excellent miscibility, resulting in a homogeneous PEDOT:PSS/PEO_8000_ matrix up to [PEO] =  37 wt %. Incorporating ultrahigh *M*_w_ PEO softens the rigid PEDOT:PSS network and increases the chain entanglements, thereby enhancing mechanical deformability (ε_break_ ∼  48%) compared to neat PEDOT:PSS films (ε_break_ ∼  5%). Moreover, at [PEO] ≥ 40 wt %, the PEO crystallization further boosts film deformability due to the semicrystalline PEO network. However, at [PEO] ≥ 50 wt %, the misorientation of adjacent spherulites disrupts the continuity of the PEDOT conductive pathways. Therefore, the PEDOT:PSS/PEO_8000_ film at [PEO] = 40 wt % gives the optimal balance of a high σ_e_ =  100 S·cm^–1^ and ε_break_ ∼  60%. In addition, the presence of flexible PEO chains not only preserves the mechanical ductility of the PEDOT:PSS conductive network under both low-humidity and high-temperature conditions but also demonstrates antifreezing capability, retaining electrical and mechanical performance even under subzero temperatures. As a result, these findings present a versatile platform for designing conductive polymer systems that operate reliably under diverse environmental conditions for next-generation wearable electronics.

## Experimental Section

### Materials

For the synthesis of PEDOT:PSS dispersion with the higher *M*_w_ PSS, see the previous study.^26^ The synthesized high-M_w_ PEDOT:PSS (PSS *M*_w_ = 1000 kg mol^–1^) have the PEDOT to PSS weight ratio of 1:2.5, and the solid contents are 0.88 wt %. The PSS with *M*_w_ = 1000 kg mol^–1^ are purchased from Scientific Polymer Products Inc. 3,4-ethylenedioxythiphene (EDOT; purity ≥ 97%) sodium persulfate (Na_2_S_2_O_8_, purity ≥ 99.0%), iron­(III) sulfate (Fe_2_(SO_4_)_3_, 97%), methanol (CH_3_OH, purity ≥ 99.0%) cation exchange resin, anion exchange resins and Polyacrylic acid (PAA; *M*_w_ = 450 kg·mol^–1^) were purchased from Sigma-Aldrich Co. and Yongin-Si, Gyeonggi-do, Korea. The poly­(ethylene oxide) (PEO) PEO_100_ (*M*_w_ = 100 kg·mol^–1^), PEO_600_ (*M*_w_ = 600 kg·mol^–1^), PEO_1000_ (*M*_w_ = 1000 kg·mol^–1^), PEO_3500_ (*M*_w_ = 3500 kg·mol^–1^), and PEO_8000_ (*M*_w_ = 8000 kg·mol^–1^) were bought from Sumitomo Chemical Co. Ltd. Polyvinylalcohol (PVA; *M*_w_ = 180 kg·mol^–1^) and dimethyl sulfoxide (DMSO) were purchased from Emperor Chemical and were used without purification.

### Preparation of the PEDOT:PSS/PEO Blend Films

To prepare the well-dispersed PEDOT:PSS solution, PEDOT:PSS solution at 0.88 wt % was given with ultrasonic treatment for 30 min. The solution was further concentrated to 2.5 wt %. After that, the PEDOT:PSS solution was blended with the PEO_8000_ aqueous solution at 2.5 wt %, followed by stirring at 50 °C overnight to prepare the homogeneous PEDOT:PSS/PEO_8000_ aqueous solution. Next, DMSO secondary dopants were added to the PEDOT:PSS/PEO_8000_ aqueous solution, and the concentration of DMSO was 2 wt %. Later, the PEDOT:PSS/PEO_8000_ solution was drop-cast onto a glass substrate and dried at 70 °C to prepare the drop-cast film. To obtain the PEDOT:PSS/PEO_8000_ free-standing blend films, the solution was poured into a PTFE mold with a size of 30 × 30 × 15 mm^3^ and then dried at 70 °C in an oven overnight. The weight content of PEO in the polymer blends was defined as the wt % = 100% × weight (PEO)/weight (PEO) + weight (PEDOT:PSS). Finally, the PEDOT:PSS/PEO_8000_ complex films were peeled off the mold and cut into a dog-bone shape of 2 × 30 mm^2^ for the mechanical and electrical conductivity tests.

### Optical Microscopy

The transmitted Optical microscopy (OM) and polarized optical microscope (POM) images of the PEDOT:PSS, PEO_8000_, and PEDOT:PSS/PEO_8000_ drop-cast films were obtained using an Olympus BX51 optical microscope.

### Wide-Angle X-ray Diffraction

Wide-angle X-ray Diffraction (WAXD) experiments for the PEDOT:PSS/PEO films were acquired at the BL13A1 and 23A1 beamlines in NSRRC. The diffraction pattern was recorded with an SX165 area detector, Mar CCD, and a CMOS flat panel detector (C9728DK), respectively. The wavelengths of the X-rays were 1.02744 Å (BL13A1) and 0.827 Å (BL23A1). The distances between samples and detectors were 180.61 mm (BL13A1) and 135.26 mm (BL23A1), giving a *q*-range of 0.2 to 2.5 Å^–1^. The scattering vector was *q*, related to the scattering angle (2θ) and the photon wavelength (λ) by *q* = 4πsin­(θ)/λ.

### Differential Scanning Calorimetry

Differential scanning calorimetry (DSC) analysis was conducted with a Hitachi DSC-7000X, Japan. The measurements were carried out under a nitrogen atmosphere, with samples scanned over a temperature range of −80 to 120 °C at a heating rate of 10 °C/min.

### Infrared Spectroscopy

Attenuated total reflection (ATR)-Fourier transform infrared (FT-IR) spectroscopy was performed on the PSS/PEO_8000_ complex films using a PerkinElmer Spectrum 3 spectrometer with a ZnSe crystal ATR attachment. The spectra were recorded through 8 scans within the 1280–780 cm^–1^ wavenumber range. Before the measurement, the PSS/PEO_8000_ complex films are dried at 100 °C overnight.

### Electrical Conductivity Characterization

The σ_e_ of PEDOT:PSS/PEO_8000_ films was determined using the conventional four-probe in-line contact method with a Keithley 2450 source meter. The thickness of the films was determined using a Leica DM2700 optical microscope.

### Humidity/Temperature-Dependent Tensile and Electrical Resistance Analysis

To evaluate the tensile behavior under different humidity conditions, the PEDOT:PSS/PEO_8000_ films were initially placed in an oven and dried at 70 °C overnight to eliminate residual water. Subsequently, the dried PEDOT:PSS/PEO samples were positioned in the Modular Force Stage (MFS; Linkam Scientific Instruments Ltd.), integrated with the Keithley 2450 source meter (SM), the liquid nitrogen cooling module (LN), and the RHGen Relative Humidity Controller (MFS-SM-LN-RHGen stage), as illustrated in Figure 5a. Before the humidity-dependent tensile tests, the dried samples were equilibrated at various RH levels of 10%, 40%, 60%, and 80% under *T* = 25 °C using the MFS-SM-LN-RHGen stage. For the temperature-dependent mechanical analysis, all samples are cooled/heated from the normal ambient condition (*T* = 25 °C; RH = 60%) to the wide temperature range (*T* = −50 and 60 °C). Following this conditioning, tensile testing was carried out at a speed of 4.8 mm·min^–1^, with the clamp gap set to 15 mm. For strain–stress curve generation, strain was determined by the ratio of the film elongation to its initial length, while stress was calculated as the applied force divided by the cross-sectional area of the measured films. The cross-sectional area (A) was determined using the equation A = width × thickness. The Young’s modulus was determined from the slope of the stress–strain curve within the linear elastic region (strain = 1%). In addition, the in situ electrical resistance variations of PEDOT:PSS/PEO_8000_ films under uniaxial tensile loading (Figure 5a) were measured at a constant strain rate of 4.8  mm·min^–1^ using the two-point probe method.

### Water Content Analysis

To obtain the water content of PEDOT: PSS-based samples, the weight change of the samples was calculated using the following equation

$$\mathrm{weight change}\;\left(\%\right)=\frac{W_{hydrated}-W_{dry}}{W_{hydrated}}\times 100\%$$

*W*_dry_ is the weight of the dried films, dehydrated in the oven at 100 °C for 5 h, and *W*_hydrated_ denotes the weight of the film after storage under specific temperature or relative humidity conditions for 10 h.

## Supplementary Material



## Notes

Synthetic procedures of PEDOT:PSS; OM images of neat PEDOT:PSS and PEO drop-cast films; Composition-dependent WAXD profiles of PEDOT:PSS/PEO samples with varying PEO *M*_w_; water content analysis of PEDOT:PSS/PEO films in the low-temperature range; OM images of PEDOT:PSS/PEO_8000_ films near the mechanical fracture under loading across wide environmental conditions; stress–strain curves and bar charts of neat PEDOT:PSS and PEDOT:PSS/PEO_8000_ films across wide environmental conditions; Stress–strain curves and bar charts of PEDOT:PSS/PVA and PEDOT:PSS/PAA films; DSC analysis of dry PVA and PAA samples (PDF)
