# Supplementary material for: Dual-Strategy Design of Molecular-Weight-Engineered PEDOT:PSS Complex Films for Enhanced Mechanical Ductility and Environmental Robustness
Source: ACS Appl Mater Interfaces. 2025 Oct 25;17(44):61315–24. doi: 10.1021/acsami.5c17154 (PMC12598696; doi:10.1021/acsami.5c17154)
Supplement: Supplementary file 1 [file am5c17154_si_001.pdf]

Supporting Information

Dual-Strategy Design of Molecular-weight Engineered PEDOT:PSS Complex Films for Enhanced Mechanical Ductility and Environmental Robustness

Jie-Dong Hu[1], Jui-Ling Shih[1], and Kuan-Yi Wu[1,*]

[1]*Department of Chemical Engineering and Biotechnology, National Taipei University of Technology, Taipei 10608, Taiwan*

***Corresponding Author**

E-mail: kywu@ntut.edu.tw

Preparation of polystyrene sulfonic acid *via* ion-exchange reaction

For the synthesis of PEDOT:PSS dispersion with the higher M_w PSS is described in the previous study.[1] As shown in **Scheme S1(a)**, sodium polystyrene sulfonate (PSSNa) with varying molecular weights (M_w = 1000 kg/mol) was dissolved in deionized water at 50 °C. After PSSNa solutions are completely dissolved, cation exchange resin was added to the solution to conduct the ion-exchange process, where the PSSNa will be protonated to polystyrene sulfonic acid (PSS).

Synthesis of PEDOT:PSS with various M_w of PSS

As shown in **Scheme S1(b)**, PEDOT:PSS aqueous solutions containing PSS with M_w =1000 kg mol^{-1} were obtained by oxidative polymerization. First, PSS was dissolved in deionized water at an approximate molar ratio of 1.9:1 to Ethylenedioxythiophene (EDOT) monomer, and degassed with nitrogen (N_2) at room temperature for one hour. The EDOT monomers were then gradually added dropwise into the reactor, stirring for 30 minutes. Subsequently, sodium persulfate ($Na_2S_2O_8$) was used as the oxidative agent at 1.5 moles per mole of EDOT, and ferric sulfate ($Fe_2(SO_4)_3$) as the catalyst at 0.02 moles per mole of EDOT. These were dissolved in deionized water and introduced slowly into the reaction mixture. The oxidative polymerization proceeded at 20 ºC for 24 hours under a N_2 atmosphere. After the reaction, the mixture was purified using cation and anion exchange resins to eliminate excess ions. The resins were subsequently filtered out, yielding a dark blue PEDOT:PSS dispersion.

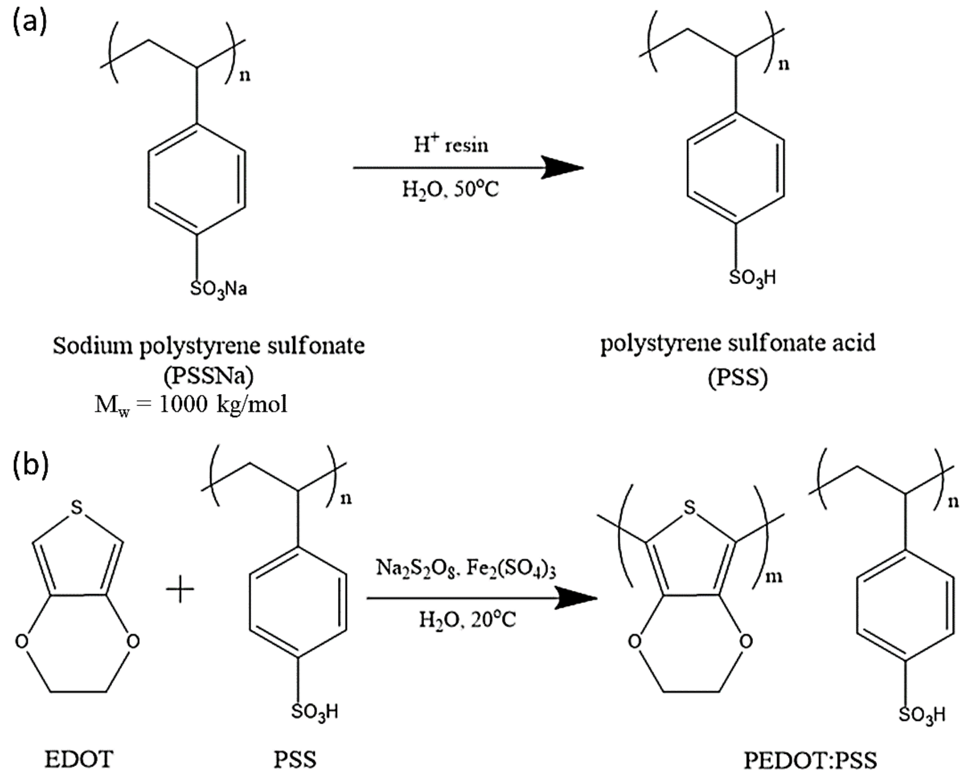

Scheme S1. (a) Cation ion-exchange reaction of PSS with M_W = 1000 kg/mol). (b) Synthetic scheme of PEDOT:PSS with M_W = 1000 kg/mol.

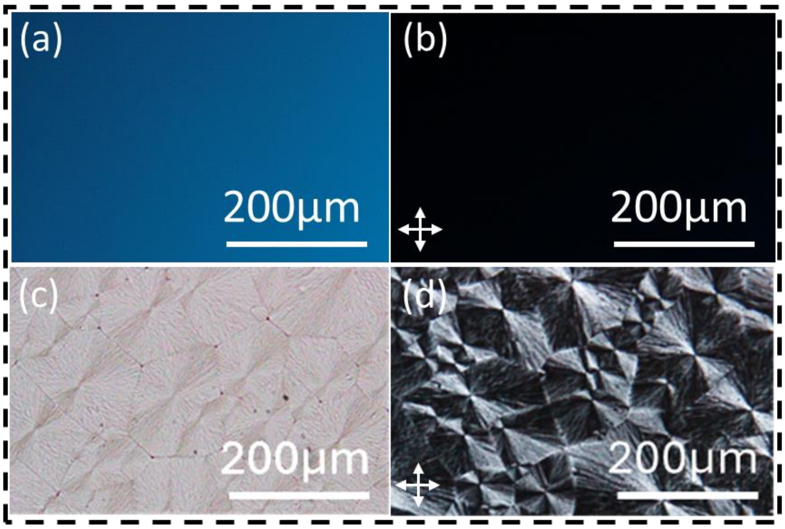

Figure S1. OM and POM micrographs of (a, b) the neat PEDOT:PSS and (c, d) PEO_{8000} drop-cast films. Note: The polarizer and the analyzer (white arrows) are in a perpendicular configuration.

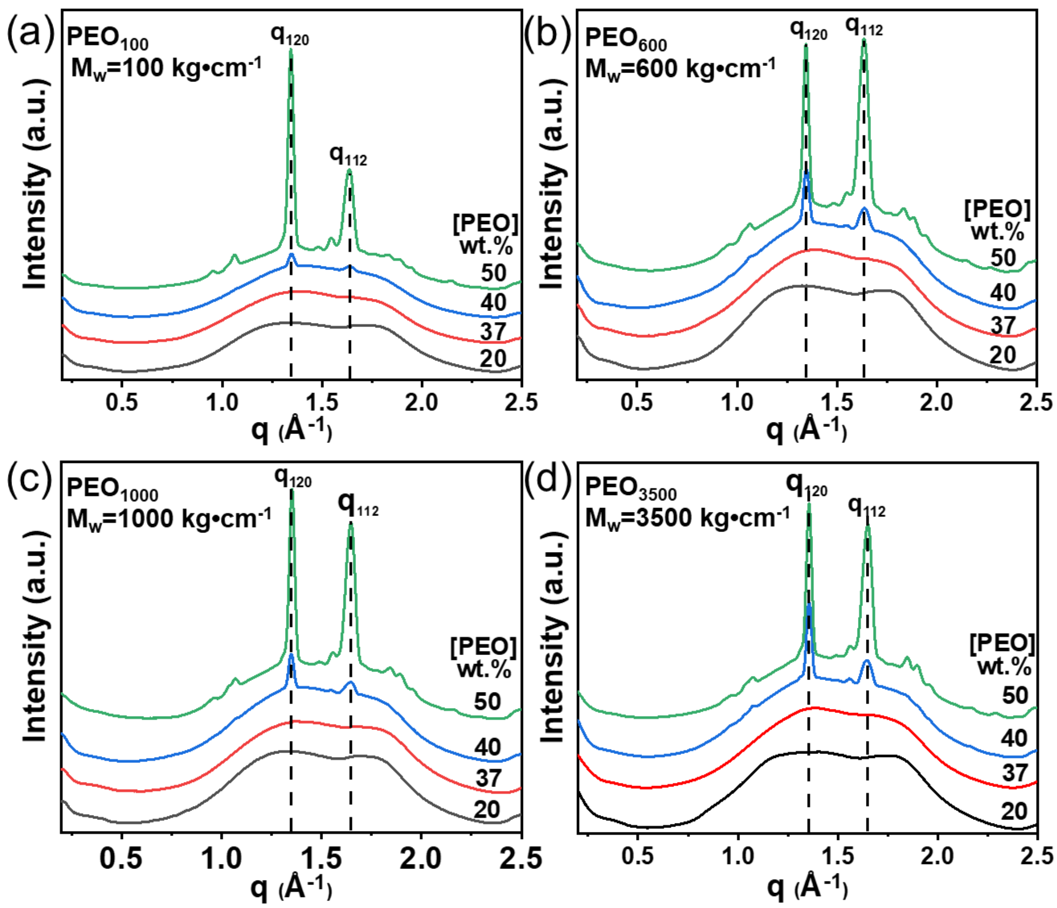

Figure S2. WAXD patterns of the PEDOT:PSS/PEO blend films with varying M_w of PEO (a) PEO100 (M_w = 100 kg·mol^{-1}), (b) PEO_{600} (M_w = 600 kg·mol^{-1}), (c) PEO_{1000} (M_w = 1000 kg·mol^{-1}), and (d) PEO_{3500} (M_w= 3500 kg·mol^{-1}).

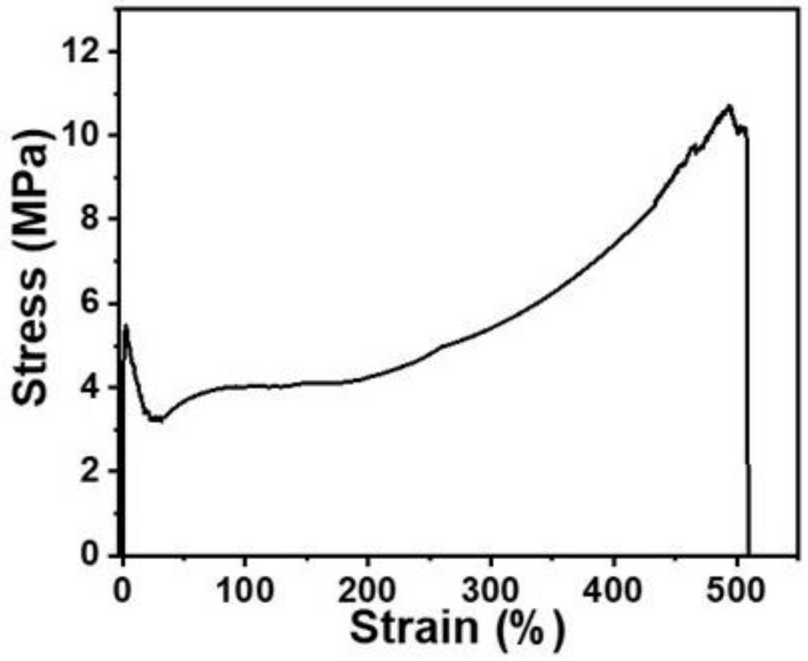

Figure S3. Strain-stress curves of PEO_{8000} film.

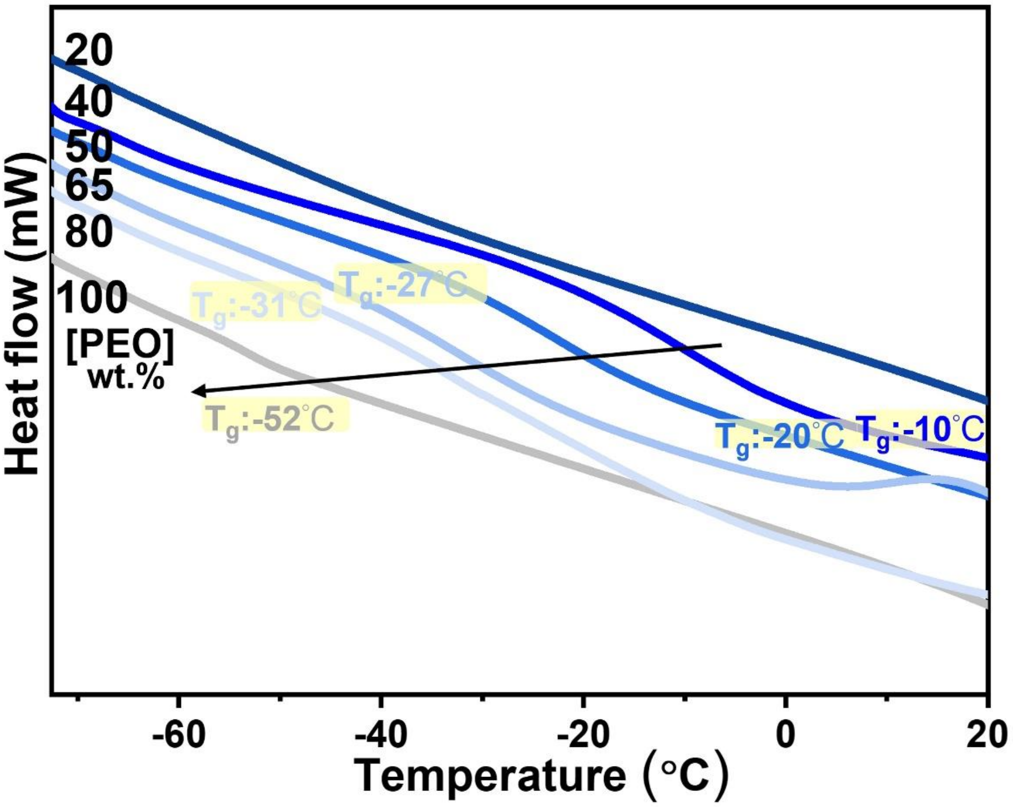

Figure S4. DSC thermograms of PEO_{8000}/PSS_{1000} film with varying PEO contents.

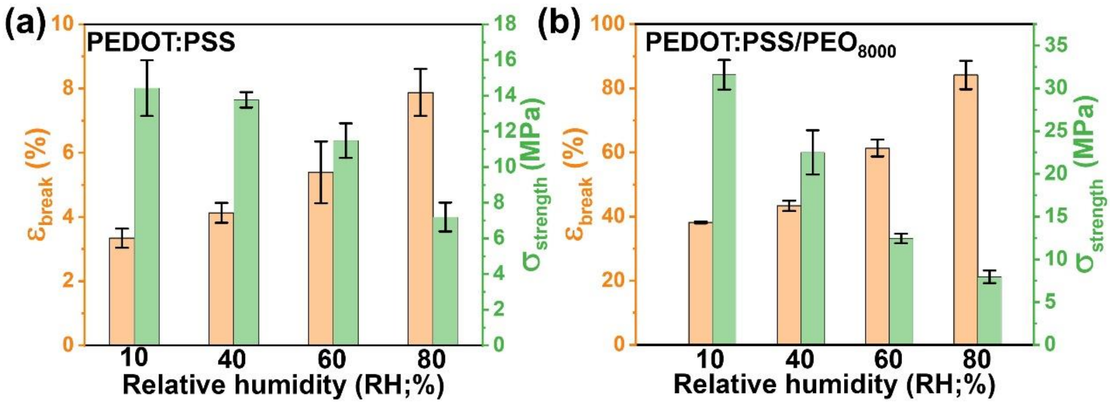

Figure S5. Tensile strength and elongations of (a) PEDOT:PSS and (b) PEDOT:PSS/PEO_{8000} films (PEO content = 40wt.%) at different RH levels under T = 25 °C. Data are expressed as mean ± SD from five independent samples (n = 5).

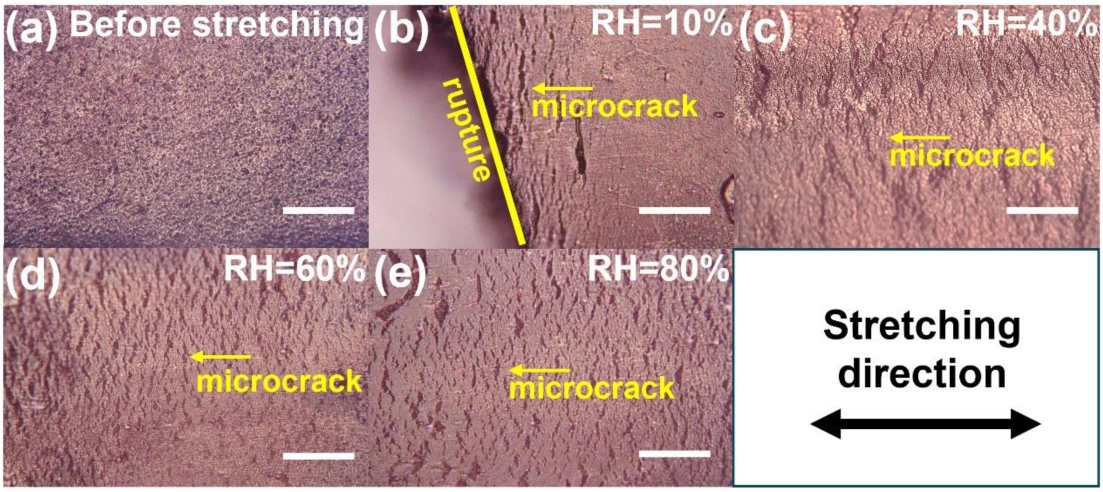

Figure S6. Reflective optical microscopy images of PEDOT:PSS/PEO_{8000} free-standing films with [PEO] = 40 wt.% at 25 °C in the wide RH ranges. (a) before the stretching process. (b-e) near the mechanical fracture (ε_{break}) under loading. Scale bar: 50μm.

Table S1. Water content in the neat PEDOT:PSS and PEDOT:PSS/PEO_{8000} (PEO content = 40 wt.%) free-standing films during the heating/cooling process from the normal ambient conditions (25 °C ; RH =60%).

**Temperature (°C)**	**-50**	**-20**	**0**	**25**	**40**	**60**
**PEDOT:PSS**		No change		20.6%	10.6%	3.2%
**PEDOT:PSS/PEO_{8000}**		No change		15.9%	6.5%	1.1%

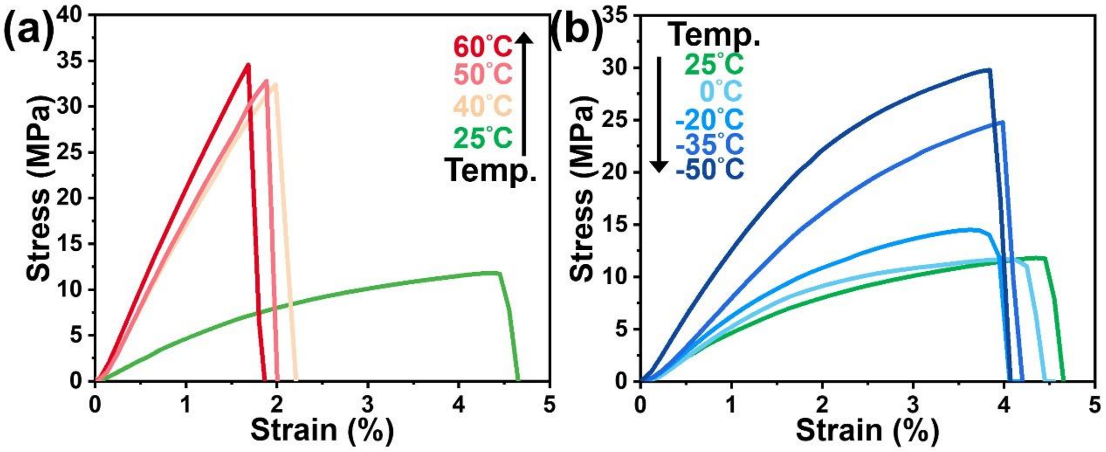

Figure S7. Tensile behavior of neat PEDOT:PSS films across a wide temperature range from (a) T = 25 °C to 60 °C and (b) 25 °C down to - 50°C.

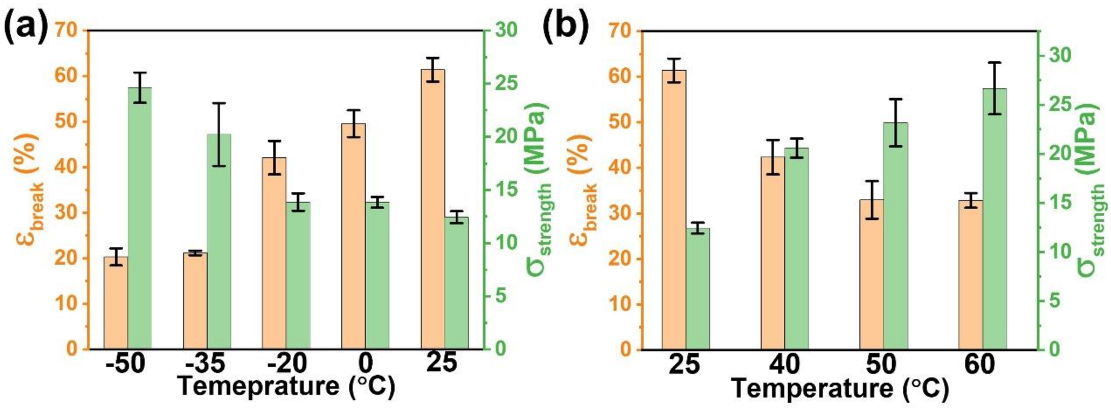

Figure S8. Tensile strength and elongations of PEDOT:PSS/PEO_{8000} films (PEO content = 40wt.%) in the wide ranges of temperatures from (a) - 50°C to 25°C and (b) 25°C to 60°C. Data are expressed as mean ± SD from five independent samples (n = 5).

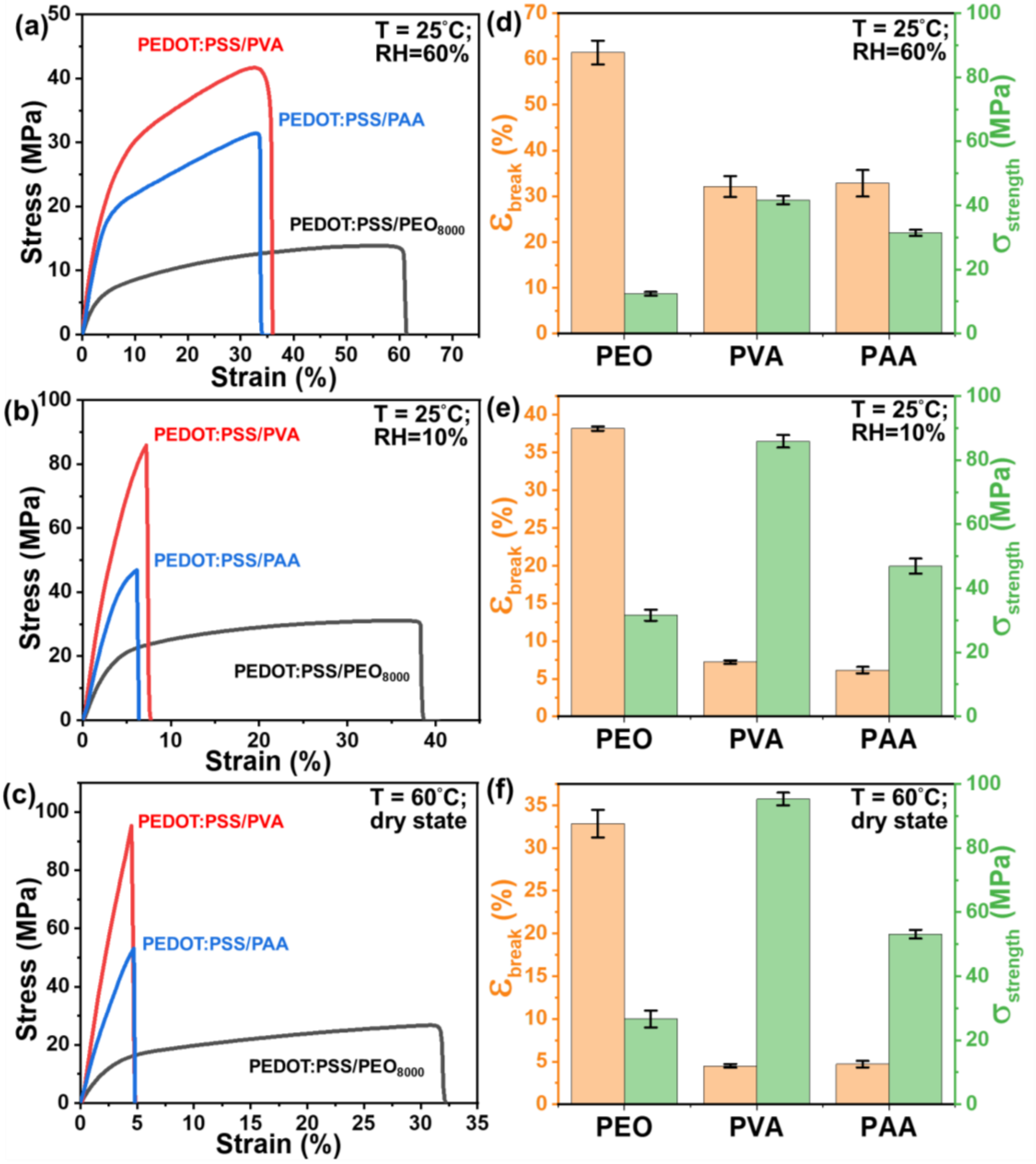

Figure S9. Comparison of (a,b,c) the tensile behavior and (d,e,f) statistical bar charts of PEDOT:PSS/PEO_{8000}, PEDOT:PSS/PVA, and PEDOT:PSS/PAA films under different environmental conditions: (a,d) T = 25°C, RH = 60%, (b,e) T = 25°C, RH = 10%, and (c,f) T = 60°C, dry state. Note: The PVA, PAA, and PEO contents in the corresponding blends are 40 wt.%. The M_w of PEO_{8000}, PVA, and PAA are 8000 kg/mol, 180 kg/mol, and 450 kg/mol, respectively.

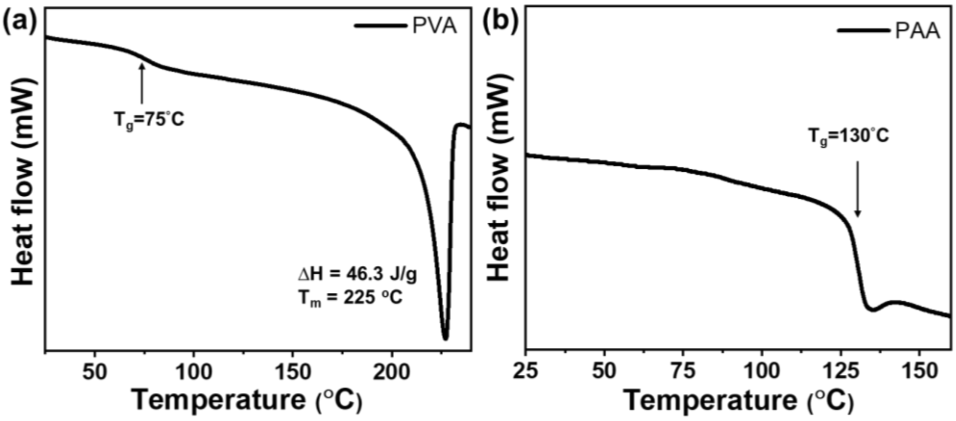

Figure S10. DSC thermograms of the (a) dry PVA and (b) PAA samples.

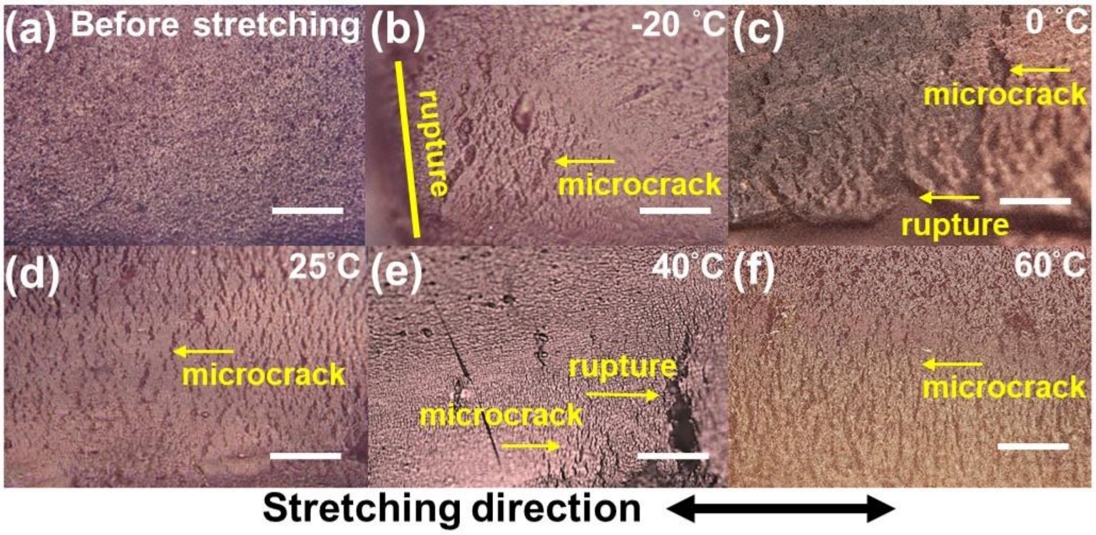

Figure S11. Reflective optical microscopy images of PEDOT:PSS/PEO_{8000} free-standing films with [PEO] = 40 wt.% in the temperature range from T = -20 °C to 60 °C. (a) before the stretching process. (b-f) near the mechanical fracture (ε_{break}) under loading. Scale bar: 50μm.

Reference

(1) Lo, H.-C.; Hu, J.-D.; Chou, C.-M.; Chen, C.-Y.; Lin, J.-M.; Chen, C.-W.; Wang, C.-L.; Chuang, W.-T.; Wu, K.-Y., Enhancing Mechanical Properties of Wet-Spun Pedot:Pss Conductive Fibers Via Molecular Weight Engineering of Pss. *Eur. Polym. J.* **2025,** *235*.
